# Trp-574-Leu and the novel Pro-197-His/Leu mutations contribute to penoxsulam resistance in *Echinochloa crus-galli* (L.) P. Beauv.

**DOI:** 10.3389/fpls.2024.1488976

**Published:** 2024-11-25

**Authors:** Penglei Sun, Liangliang Niu, Pengfei He, Haiyan Yu, Jingchao Chen, Hailan Cui, Xiangju Li

**Affiliations:** Institute of Plant Protection, Chinese Academy of Agricultural Sciences, Beijing, China

**Keywords:** *Echinochloa crus-galli*, mutation, penoxsulam, molecular docking, resistance

## Abstract

Recently, due to the widespread use of the acetolactate synthase (ALS)-inhibiting herbicide penoxsulam in paddy fields in China, *Echinochloa crus-galli* (L.) P. Beauv. has become a problematic grass weed that is frequently not controlled, posing a threat to weed management and rice yield. There are many reports on target-site mutations of ALS inhibiting herbicides; however, the detailed penoxsulam resistance mechanism in *E. crus-galli* remains to be determined. Greenhouse and laboratory studies were conducted to characterize target-site resistance mechanisms in JL-R, AH-R, and HLJ-R suspected resistant populations of *E. crus-galli* survived the field-recommended dose of penoxsulam. The whole-plant dose–response testing of *E. crus-galli* to penoxsulam confirmed the evolution of moderate-level resistance in two populations, JL-R (9.88-fold) and HLJ-R (8.66-fold), and a high-level resistance in AH-R (59.71-fold) population. *ALS* gene sequencing identified specific mutations in resistant populations, including Pro-197-His in *ALS1* for JL-R, Trp-574-Leu in *ALS1* for AH-R, and Pro-197-Leu in *ALS2* for HLJ-R. *In vitro* ALS activity assays demonstrated a significantly higher activity in AH-R compared to the susceptible population (YN-S). Molecular docking studies revealed that Trp-574-Leu mutation primarily reduced the enzyme’s ability to bind to the triazole-pyrimidine ring of penoxsulam due to decreased π–π stacking interactions, while Pro-197-His/Leu mutations impaired binding to the benzene ring by altering hydrogen bonds and hydrophobic interactions. Additionally, the Pro-197-His/Leu amino acid residue changes resulted in alterations in the shape of the active channel, impeding the efficient entry of penoxsulam into the binding site in the ALS protein. The three mutant ALS proteins expressed via the Bac-to-Bac baculovirus system exhibited notably lower activity inhibition rates than the non-mutant ALS proteins to penoxsulam, indicating all three ALS mutations reduce sensitivity to penoxsulam. This study elucidated the distinct impacts of the Pro-197-His/Leu and Trp-574-Leu mutations in *E. crus-galli* to penoxsulam resistance. Notably, the Trp-574-Leu mutation conferred stronger resistance to penoxsulam compared to the Pro-197-His/Leu mutations in *E. crus-galli*. The Pro-197-His/Leu mutations were first detected in *E. crus-galli* conferring penoxsulam resistance. These findings provide deeper insights into the molecular mechanisms underlying target-site resistance to penoxsulam in *E. crus-galli.*

## 1 Introduction

Weed infestation poses the most significant biotic threat to global food security, leading to yield reductions of up to 34% in agriculture (Oerke, 2006). With their pervasive presence in virtually every crop field, effective management and control of these weeds are imperative to ensure high crop yields. A variety of methods are available for weed control in agricultural fields, including manual and mechanical techniques, chemical control, crop rotation, and biological approaches (Adkins and Shabbir, 2014; Latif et al., 2015). Synthetic chemical herbicides serve as the primary tools employed worldwide (Brun et al., 2022). The application of herbicides with different modes of action has facilitated weed control, playing an important role in yields and qualities of crops (Kraehmer et al., 2014). Among these, acetolactate synthase (ALS, EC 4.1.3.18) inhibitors possess several distinctive characteristics in agricultural applications. These features encompass low application quantities, broad spectrum, expansive application windows, and low mammalian toxicity, coupled with high crop safety margins (Mazur and Falco, 1989; Singh et al., 2019).

Acetolactate synthase, also known as acetohydroxyacid synthase (AHAS, EC 2.2.1.6), plays a pivotal role as an enzyme in the biosynthesis of branched-chain amino acids, such as leucine, isoleucine, and valine in plants (Devine and Shukla, 2000; Duggleby et al., 2008). ALS can catalyze the conversion of two molecules of pyruvate into 2-acetolactate and the transformation of one molecule of 2-ketobutyrate and one molecule of pyruvate into 2-aceto-2-hydroxybutyrate (Duggleby et al., 2008; Zhou et al., 2007). The ALS enzyme consists of a catalytic subunit and a regulatory subunit, with necessary cofactors including thiamine diphosphate (ThDP or TPP), flavin adenine dinucleotide (FAD), and divalent metal ion (Mg^2+^) for the catalytic activity of the catalytic subunit (Chipman et al., 1998). ALS inhibitor herbicides are primarily categorized into five groups based on differences in the chemical structure of compounds: triazolopyrimidines (TPs), imidazolinones (IMIs), sulfonylureas (SUs), pyrimidinyl-thiobenzoates (PTBs), and sulfonyl-aminocarbonyl-triazolinones (SCTs). However, the excessive use of these chemicals has led to the evolution of herbicide-resistant weeds. Up to now, 174 weed species developed resistance to ALS inhibitors globally (Heap, 2024).

Herbicide resistance mechanisms are broadly categorized into target-site resistance (TSR) and nontarget-site resistance (NTSR) (Comont et al., 2020; Délye, 2013; Délye et al., 2013; Gaines et al., 2020). TSR involves the mutation of amino acids in the target enzyme through gene mutations or variations in gene copy numbers (Pan et al., 2022). A total of 24 amino acid sites are associated with resistance to ALS inhibitors in weeds, yeast, and bacteria (Yu and Powles, 2014). In relation to ALS inhibitors, various weed species have reported 31 types of amino acid mutations at nine conserved positions (Ala-122, Pro-197, Ala-205, Phe-206, Asp-376, Arg-377, Trp-574, Ser-653, and Gly-654), numbered according to the corresponding *Arabidopsis thaliana* (L.) Heynh. Sequence (Fang et al., 2022; Li et al., 2022; Powles and Yu, 2010; Tranel et al., 2024). Various mutations in the *ALS* gene have been demonstrated to confer distinct resistance patterns in weed populations (Yu and Powles, 2014). Mutations occurring at different amino acid positions can grant varying degrees of resistance within the same weed species (Cao et al., 2022; Fang et al., 2019b; Riar et al., 2013; Wang et al., 2021). Additionally, different mutations of the same amino acid can result in diverse cross-resistance patterns (Deng et al., 2014). It is noteworthy that the same mutation may lead to differing resistance in different weed species (Li et al., 2022). These resistance patterns are intricately tied to the structure of the ALS protein and the properties of the mutated amino acids. Various *ALS* gene mutations can accumulate within individual plants through cross-pollination, as evidenced by the simultaneous identification of the Pro-197-Thr and Trp-574-Leu mutations in individual *Descurainia sophia* L. plants (Deng et al., 2017). Accumulation of mutations within the same weed species may confer resistance to multiple herbicides, thereby adding complexity to the challenges of weed resistance management.

*Echinochloa crus-galli* (L.) P. Beauv., a pervasive weed in paddy rice (*Oryza sativa* L.) fields worldwide, has similar morphology and growth habits with rice and is difficult to be recognized and identified especially in its seedling stage. Being a C4 plant, it competes strongly in photosynthesis and may act as an intermediary host for certain pests and diseases, significantly affecting rice yield and quality (Chauhan and Johnson, 2011; Zhang et al., 2017). The ALS inhibitor penoxsulam is commonly used for post-emergence weed control, especially against *E. crus-galli* in rice field, in various types of culture, methods of planting, and cultivars of the crop in China. However, the overreliance on penoxsulam has led to the development of resistant weeds related to TSR and NTSR (Fang et al., 2019a, Fang et al., 2022, Fang et al., 2019b). To date, 12 different *ALS* gene mutations have been reported to endow resistance to ALS inhibitors in *Echinochloa* spp. (**Table 1**) for TSR mechanism (Amaro-Blanco et al., 2021; Délye et al., 2015; Fang et al., 2019a, Fang et al., 2022, Fang et al., 2019b; Feng et al., 2022; Liu et al., 2019; Panozzo et al., 2017; Tranel et al., 2024). Among these, mutations at positions Pro-197 and Trp-574 have commonly been documented in resistant weeds (Tranel et al., 2024). Clear evidence has shown that Trp-574-Arg/Leu mutations confer penoxsulam resistance in *E. crus-galli*, whereas mutations at position 197 have not been reported to confer penoxsulam resistance in *E. crus-galli* (Fang et al., 2019b; Feng et al., 2022).

**Table 1:** Different amino acid mutations at *ALS* sites.

Site	Amino acid mutation	Site	Amino acid mutation
122	Ala→Gly/Val/Thr/Asn/Ser/Tyr	377	Arg→His
197	Pro→Ser/Thr/Leu/His/Arg/Gln/Ala/Ile/Asn/Tyr	574	Trp→Arg/Leu/Gly/Met
205	Ala→Val/Phe	653	Ser→Asn/Thr/Ile
206	Phe→Leu/Tyr	654	Gly→Glu/Asp
376	Asp→Glu		

Highlighted in red were the types of ALS mutations that had been reported in resistant *Echinochloa* spp. Highlighted in blue is a novel mutation in the *E. crus-galli* ALS gene reported in this report.

The objective of this study is to fully investigate the TSR mechanism based on the suspected populations of *E. crus-galli* collected from rice fields in China, aimed to (1) demonstrate the resistance levels among different mutation populations to penoxsulam, (2) explore the underlying mechanisms of TSR, and (3) clarify the distinctions in resistance between Pro-197-His/Leu and Trp-574-Leu mutations.

## 2 Materials and methods

### 2.1 Plant materials and herbicide

In this research, four populations of *E. crus-galli* were investigated, each distributed in different geographical regions from China. The phenotypically resistant populations, JL-R (Tonghua city, Jilin province; 42.62°N, 126.07°E), AH-R (Hefei city, Anhui province; 31.25°N, 117.20°E), and HLJ-R (Hegang city, Heilongjiang province; 47.50°N, 130.86°E), were collected from rice fields where penoxsulam had proven ineffectiveness in weed control. In contrast, the susceptible population, YN-S (coordinates 30.91°N, 118.80°E), was obtained from a non-cultivated field close to rice paddies in Yunnan Province, where no herbicides had been previously used. For each of these four *E. crus-galli* populations, seeds were collected from a minimum of 40 individual plants and stored in a well-ventilated shelf. Penoxsulam (2-(2,2-difluoroethoxy)-N-(5,8-dimethoxy-[1,2,4]triazolo[1,5-c]pyrimidin-2-yl)-6-(trifluoromethyl)benzenesulfonamide, 25 g/L oil dispersion) was obtained from Dow AgroSciences.

### 2.2 Whole-plant dose–response bioassay

Seeds from each of the JL-R, AH-R, HLJ-R, and YN-S populations were randomly selected and germinated in Petri dishes with damp filter papers, then incubated in a growth chamber with a temperature of 30°C/25°C and a 12-h photoperiod. After the shoot length reached approximately 1 cm, seven seedlings were transplanted into four replicate pots filled with a commercial potting soil mixture for each herbicide rate. These pots were placed in a growth chamber set at 30°C/25°C with a 12-h day/night cycle. Penoxsulam was applied at the rates 0 g a.i. ha^−1^, 1.67 g a.i. ha^−1^, 5 g a.i. ha^−1^, 15 g a.i. ha^−1^, 45 g a.i. ha^−1^, 135 g a.i. ha^−1^, and 405 g a.i. ha^−1^ for the JL-R and HLJ-R populations; 0 g a.i. ha^−1^, 5 g a.i. ha^−1^, 15 g a.i. ha^−1^, 45 g a.i. ha^−1^, 135 g a.i. ha^−1^, 405 g a.i. ha^−1^, and 1,215 g a.i. ha^−1^ for the AH-R population; and 0 g a.i. ha^−1^, 0.19 g a.i. ha^−1^, 0.56 g a.i. ha^−1^, 1.67 g a.i. ha^−1^, 5 g a.i. ha^−1^, 15 g a.i. ha^−1^, and 45 g a.i. ha^−1^ for the YN-S population at three- to four-leaf stages of *E. crus-galli* using an experimental moving-boom sprayer (Model ASS-4, Beijing Research Center for Information Technology in Agriculture, China), equipped with a TeeJet XR8002VS flat-fan nozzle and a pressure of 0.275 MPa, delivering the volume of 450 L ha^−1^ liquid. *E. crus-galli* growing in the pots were all harvested, and their dry weight biomass (after 3 days of 75°C oven-dried) was measured 3 weeks after herbicide treatment. The whole-plant dose–response experiment was conducted twice.

### 2.3 *ALS*-cDNA and *ALS*-DNA sequencing

Seeds were cultured to three- to four-leaf stage as described in *Section 2.2*. Young plant leaf tissue, weighing 100 mg for each, was sampled from a minimum of 20 plants of each population. DNA was extracted using the DNAsecure Plant Kit (TianGen Biotech, Co. Ltd., Beijing, China), and total RNA was isolated using the RNA Easy Fast Plant Tissue Kit (TianGen Biotech, Co. Ltd., Beijing, China). First-strand cDNA was synthesized using the *HiScript* ^®^ III 1st Strand cDNA Synthesis Kit (Vazyme Biotech Co., Ltd., Nanjing, China).

*E. crus-galli*, an allohexaploid grass weed, harbors at least three *ALS* genes (Fang et al., 2022; Iwakami et al., 2015; Panozzo et al., 2021). Primers ALS-1 (forward, 5′-ATCCCCCATCCTCTCCTT-3′; reverse, 5′-GGTCCAGAGTTCACACCCTAG-3′) and primers ALS-2 (forward, 5′-CACCCTCCCCAAACCC-3′; reverse, 5′-CACGGAAACAACAGACTACAT-3′) were designed based on the *ALS* mRNA sequences of *E. crus-galli LC006058.1* and *LC006059.1* to amplify the complete *ALS1* and *ALS2* genes. Additionally, primers ALS-3 (forward, 5′-CCCCAATCCCCCATCCAT-3′; reverse, 5′-GCACCGCTCGCTGAATAC-3′) reported by Iwakami and coworkers were used to specific amplification of *ALS3* gene (Iwakami et al., 2015). The fragments of *ALS1-3* cover the known eight resistance-conferring mutation sites. For polymerase chain reaction (PCR), LA Taq^®^ was used with GC Buffer (Takara Biomedical Technology Co., Ltd., Beijing, China) according to the manufacturer’s instructions. The purification and TA cloning of the PCR products were conducted using the methods outlined in a previous report (Sun et al., 2023). A minimum of eight white colonies were carefully selected and then sent to Tsingke Biotechnology Co., Ltd. (Beijing, China) for Sanger sequencing. The resulting sequences were aligned with the documented *ALS* genes of *E. crus-galli* (accession numbers, *LC006058.1*, *LC006059.1*, and *LC006063.1*) using NCBI-BLAST and analyzed using DNAMEN 9.0.1 (Lynnon Corporation, Quebec, Canada) and SeqMan Pro (v7.1.0, DNASTAR Lasergene) (Clewley, 1995).

### 2.4 *In vitro* assay of ALS activity

The extraction and evaluation of the ALS enzyme were conducted following the methods outlined by Yu with coworkers (Yu et al., 2004) and Han with coworkers (Han et al., 2012) with minor modifications. *E. crus-galli* seedlings (purified and ensured to carry the special mutation for all plants) were cultivated to the three- to four-leaf stage following the aforementioned method. Approximately 4 g of leaf tissue was sampled from each population, with a minimum of 30 seedlings harvested in each sample. The frozen leaf material was homogenized using a mortar and pestle in 8 mL of grinding buffer. Subsequently, an equal volume of 100% (NH4)_2_SO_4_ was added dropwise to the solution, followed by centrifugation. The resulting ALS pellet was redissolved in 3.5 mL resuspension buffer. ALS protein was desalted using a Sephadex G25 column with a 5-mL elution buffer.

For the enzyme assays, 100 μL of desalted enzyme extraction solution and 100 μL of penoxsulam at different concentrations were added to a 1.5-mL centrifugation tube. The dosage of penoxsulam was set at 0 μmol·L^−1^, 0.00001 μmol·L^−1^, 0.0001 μmol·L^−1^, 0.001 μmol·L^−1^, 0.01 μmol·L^−1^, 0.1 μmol·L^−1^, 1 μmol·L^−1^, 10 μmol·L^−1^, and 100 μmol·L^−1^ for YN-S population, while it was set at 0 μmol·L^−1^, 0.0001 μmol·L^−1^, 0.001 μmol·L^−1^, 0.01 μmol·L^−1^, 0.1 μmol·L^−1^, 1 μmol·L^−1^, 10 μmol·L^−1^, 100 μmol·L^−1^, and 1,000 μmol·L^−1^ for the JL-R, AH-R, and HLJ-R populations, respectively. The ALS enzyme concentration was determined using the Easy Protein Quantitative Kit (Bradford) (TransGen Biotech, Beijing, China), and the absorbance at 530 nm was measured using the FlexStation 3 full-wavelength scanning multifunctional enzyme marker (MD Electronics, USA) to determine ALS enzyme activity. The assay was conducted twice, with each treatment tested in three replicates.

### 2.5 *ALS* gene expression

The F1 generation of bagged selfed seeds from *E. crus-galli* mutated plants were all harvested for this experiment. After *E. crus-galli* seedlings reached three- to four-leaf stage, each tested population was divided into two subgroups. One subgroup was treated with 15 g a.i. ha^−1^ penoxsulam, while the other was treated with water. The leaf tissues were collected at 0 d, 1 d, 2 d, and 3 d after treatment with 10 plants sampled each day, respectively. The samples were immediately flash frozen in liquid nitrogen. Total RNA extraction and first-strand cDNA was synthesized as previously described. The *ALS* gene and *β-actin* reference gene were according to previous reports (Fang et al., 2022, Fang et al., 2019b). qRT-PCR was conducted following the manufacturer’s instructions for Taq Pro Universal SYBR qPCR Master Mix (Vazyme Biotech Co., Ltd., Nanjing, China), and the qRT-PCR program was executed using an ABI 7500 Fast Real-Time PCR System (Applied Biosystems, Waltham, MA, USA). The relative quantification of the *ALS* gene was calculated using the 2^−ΔΔCt^ method, where ΔCT =ALS (Mean CT) − Actin (Mean CT), ΔΔCT = ΔCT (treatment) − ΔCT (control) (Bustin et al., 2009; Livak and Schmittgen, 2001). ALS expression level was normalized to the Ct values for the YN-S population at 0 d (water treatment). Each process was repeated three times. Independent sample T-test was performed using SPSS v25.0 (IBM, Armonk, NY, USA) software to determine significant differences in the expression levels.

### 2.6 Homology modeling and molecular docking

The *ALS* gene sequence alignment revealed three different mutations in the populations of *E. crus-galli*: JL-R (Pro-197-His, ALS1), AH-R (Trp-574-Leu, ALS1), and HLJ-R (Trp-197-Leu, ALS2). The amino acid sequences of these three mutated proteins, along with two non-mutated proteins (listed in **Supplementary Table S1**), were employed as query templates to the homology model using SWISS-MODEL (https://swissmodel.expasy.org/) (Waterhouse et al., 2018). The ALS protein (PDB ID 3e9y) from *A. thaliana* was used as the template protein. The homology modeling results were uploaded to SAVES (http://services.mbi.ucla.edu/SAVES/) to evaluate by PROCHECK. The chemical structure of penoxsulam was downloaded from the PubChem database (CID: 11784975) (https://pubchem.ncbi.nlm.nih.gov/). Molecule docking simulations were performed by Autodock Vina (Trott and Olson, 2010), according to previously reported method with a slight modification (Butt et al., 2020). The docking box was positioned at the active site, as reported in previous literature (Fang et al., 2022; Wang et al., 2009), where the protein bound with the penoxsulam ligand. The box size was set to 15 Å × 15 Å × 15 Å, with the active site coordinates as follows: center_x = 55.026, center_y = 50.923, and center_z = 46.274. The parameter settings were exhaustiveness = 400 and num_modes = 20, and other parameters were set to their default values.

### 2.7 Heterologous expression of ALS protein and activity assay

Three mutant and two non-mutant ALS protein-coding sequences (CDS) were codon optimized, synthesized, and confirmed through Sanger sequencing by Sangon (Sangon Biotech (Shanghai) Co., Ltd.). The Bac-to-Bac baculovirus expression system and the purification of the five target proteins were conducted following the methods described in previous literature (Fang et al., 2022). The expression vector for the target gene was constructed based on Fang’s method and transformed into DH5α cells for amplification. Plasmid extraction and PCR verification were performed to confirm correctness. The plasmid was then transferred into DH10Bac competent cells via a transposition reaction, following the instructions of the Bac-to-Bac^®^ TOPO^®^ Expression System (Thermo Fisher Technology Co. Ltd, Shanghai, China). SF9 cells were inoculated at a density of 1 × 10^6^ cells mL^−1^ in 1-L culture flasks, totaling 200 mL of cells. To infect the cells, 0.8 mL of P4 viral stock was added to the 200-mL cell suspension. After 3 d, cells were collected and centrifuged at 4°C. The supernatant was then collected for ALS protein purification. The quantification of the expressed ALS proteins (diluted in elution buffer) was performed as mentioned above. The protocol used for the *in vitro* heterologous ALS activity assay closely resembled that for ALS isolated from *E. crus-galli*, with the exception that in the former case, the penoxsulam concentration was set at 1 μM. Each treatment was performed with three replicates.

### 2.8 Data analysis

The whole-plant dose–response bioassay data were subjected to a four-parameter log-logistic equation analysis using SigmaPlot 12.5 software (Systat Software, Inc., San Jose, CA, USA) to calculate the GR_50_ value (the dose causing a 50% reduction in above-ground dry weight). The regression equation used is as follows:


$$y=C+\left(D-C\right)/[1+{\left(x/GR_{50}\right)}^{b}]$$

In this equation, *y* represents the ratio of dry weight at *x* herbicide dose to that of the control, x represents the herbicide treatment dose, C represents the lower limit of the dose–response, D represents the upper limit of the dose–response, and *b* denotes the slope of the curve.

Based on the GR_50_ values obtained for each population, the resistance index (RI) was calculated using the following formula:


$$\text{RI}=\frac{{\text{GR}}_{50}\text{ of resistant population}}{{\text{GR}}_{50}\text{ of susceptible population}}$$

The same analysis calculated the herbicide concentration required to inhibit 50% of ALS activity (I_50_) and its corresponding RI value. The resistance classification criteria were as follows: RI < 2, indicating a susceptibility; 2 ≤ RI < 5, indicating a low-level resistance; 5 ≤ RI < 10, indicating a moderate-level resistance; and RI > 10, indicating a high-level resistance.

## 3 Results

### 3.1 Whole-plant dose–response to penoxsulam

**Figure 1:**
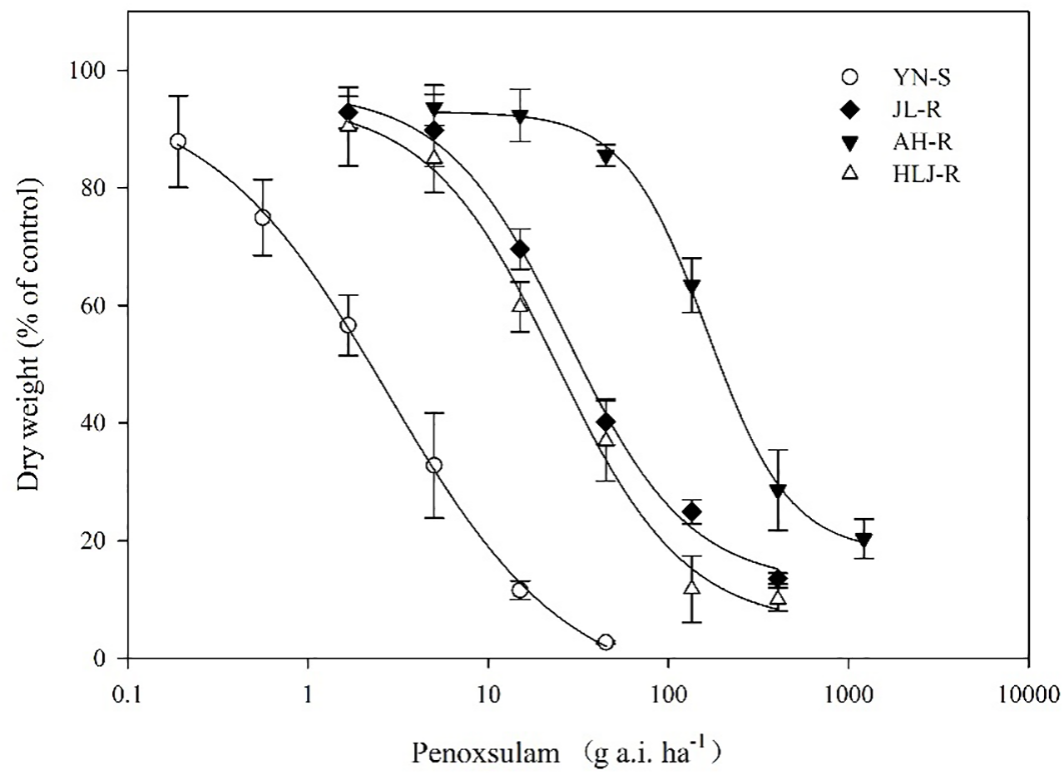
Dose–response curves for dry weights of YN-S, JL-R, AH-R, and HLJ-R populations of *Echinochloa crus-galli* to penoxsulam.

All seedlings of the S population, YN-S, were completely inhibited at 15 g a.i. ha^−1^ penoxsulam with a GR_50_ value of 2.76 g a.i. ha^−1^. The GR_50_ values of the JL-R, HLJ-R, and AH-R populations (R) were 27.26 g a.i. ha^−1^, 23.91 g a.i. ha^−1^, and 164.81 g a.i. ha^−1^, respectively, corresponding to R/S GR_50_ ratios (RI) of 9.88-fold, 8.66-fold, and 59.71-fold (**Table 2**; **Figure 1**) compared to the YN-S population, which revealed that the JL-R and HLJ-R populations had evolved moderate-level resistance and AH-R population showed high-level resistance to penoxsulam.

**Table 2:** The resistance levels of YN-S, JL-R, AH-R, and HLJ-R populations with distinct mutations of *Echinochloa crus-galli* to penoxsulam.

Population	Mutation	GR_50_ (SE)^a^	RI^b^
YN-S	None	2.76 (0.28)	1.00
JL-R	ALS1_Pro-197-His	27.28 (3.78)	9.88
AH-R	ALS1_Trp-574-Leu	164.81 (9.96)	59.71
HLJ-R	ALS2_Pro-197-Leu	23.91 (4.89)	8.66

a GR_50_ refers to the effective herbicide dose (g a.i. ha^−1^) causing 50% inhibition of dry weight; The data were the means of two experiments; SE, standard error.

b RI, resistance index

### 3.2 *ALS* gene sequencing and comparison

**Figure 2:**
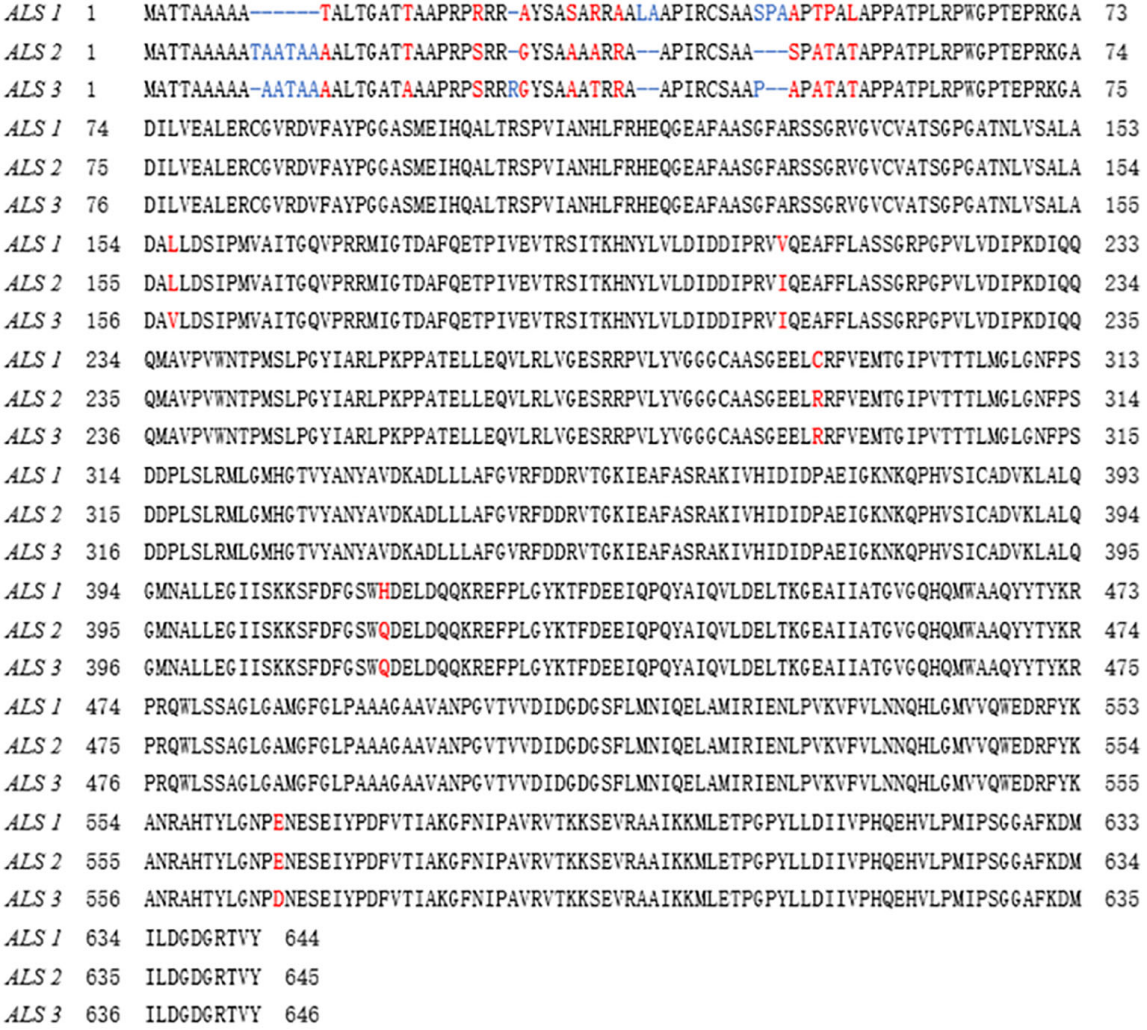
Alignment of the whole amino acid sequences of the three *ALS* copies in *E. crus-galli*.

**Figure 3:**
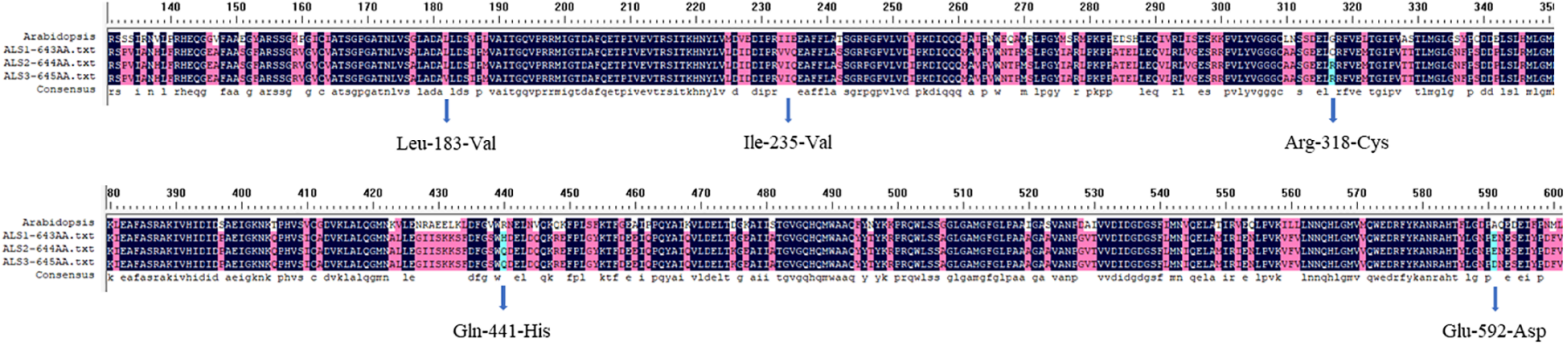
Alignment of the amino acid sequences of three *E. crus-galli ALS* copies with localization based on *Arabidopsis thaliana ALS* amino acid positions.

**Figure 4:**
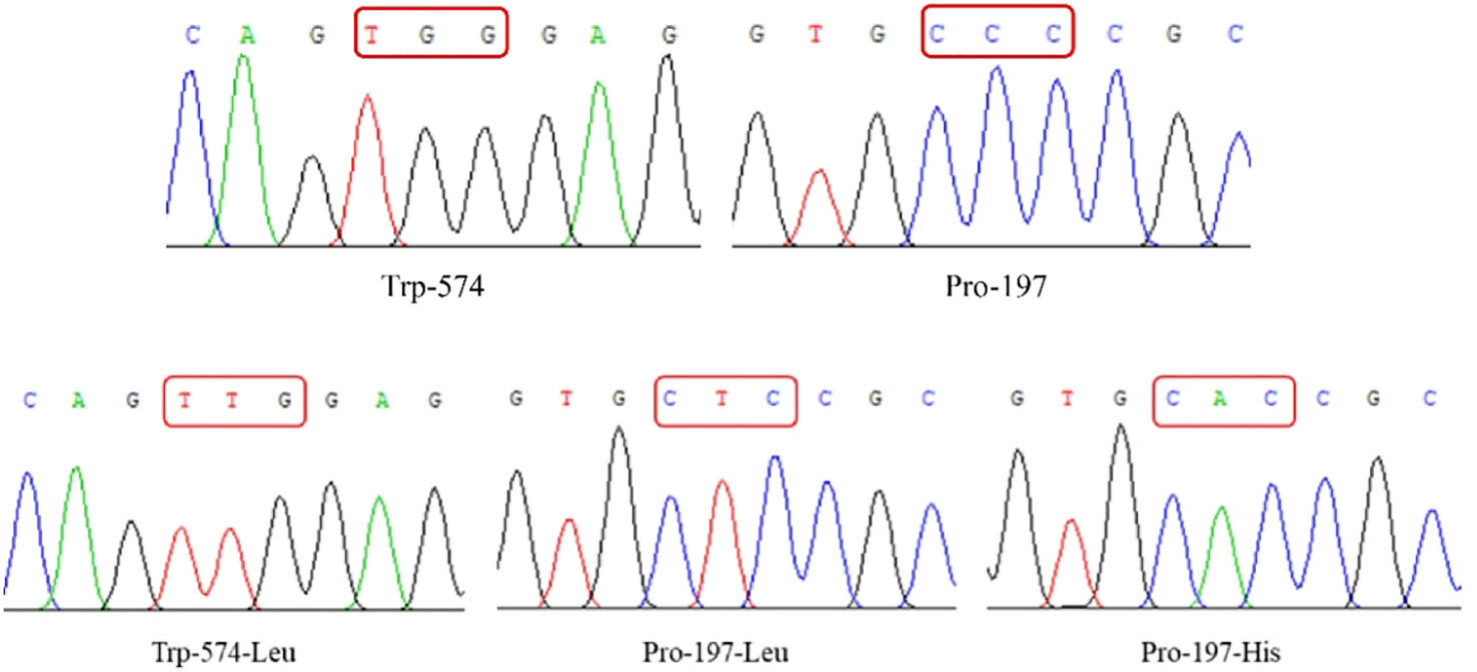
The spectrum of Trp-574-Leu and Pro-197-Leu/His mutations. The wild-type ALS gene is Leu at position 574 and Pro at position 197.

Sequencing confirmed that the *ALS* genes in *E. crus-galli* have no introns. Three *ALS* gene sequences were obtained, with lengths of 1,929 bp (*ALS1*), 1,932 bp (*ALS2*), and 1,935 bp (*ALS3*), encoding 643, 644, and 645 amino acids, respectively. Furthermore, sequence alignment analysis conducted using the COBALT tool on NCBI (https://www.ncbi.nlm.nih.gov/tools/cobalt/) identified five conserved amino acid mutations at specific positions within three distinct *ALS* copies (**Figures 2**, **3**). Three nonsynonymous nucleotide mutations were identified in three resistant *E. crus-galli* populations. The Pro-197-His (CCC to CAC), Trp-574-Leu (TGG to TTG), and Pro-197-Leu (CCC to CTC) mutations caused by a nucleotide mutation were detected in *ALS1* in JL-R population, in *ALS1* in AH-R population, and in *ALS2* in HLJ-R population, respectively (**Figure 4**). The mutation frequency in all three populations was 100%, and all colonies tested from each population showed consistent results. Additionally, Pro-197-His/Leu mutations were first identified in penoxsulam resistant *E. crus-galli*, with Pro-197-His mutation being reported for the first time in *Echinochloa* spp.

### 3.3 *In vitro* assay of ALS activity

**Figure 5:**
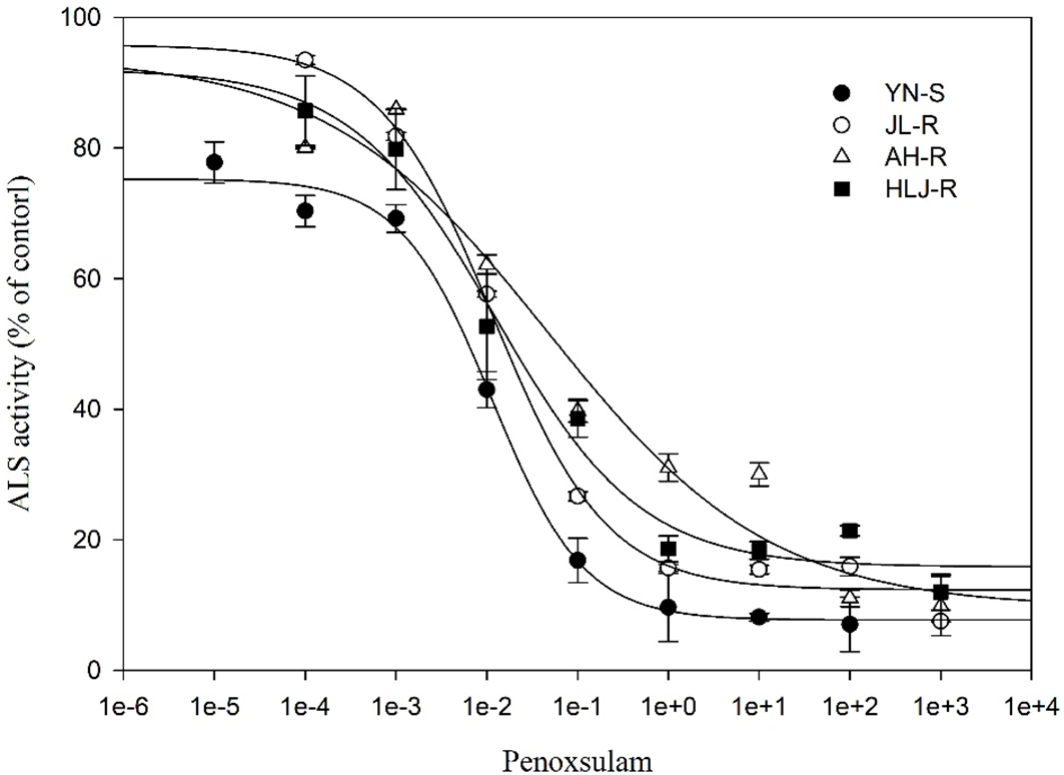
ALS activity *in vitro* of *E. crus-gulli*.

The *in vitro* assay of *E. crus-galli* ALS activity showed no significant differences (*p* > 0.05) in total catalytic activity between the resistant populations JL-R (Pro-197-His), AH-R (Trp-574-Leu), and HLJ-R (Pro-197-Leu) compared to the susceptible YN-S population (**Table 3**). As shown in the dose–response curves (**Figure 5**), when the penoxsulam dose was 0.01 μmol·L^−1^, the ALS activity in the YN-S population was significantly inhibited, with an inhibition rate exceeding 50%, while the R populations showed less inhibition. Specifically, the I_50_ value for JL-R and HLJ-R population was 0.0118 μmol·L^−1^ and 0.0126 μmol·L^−1^, only 1.04- and 1.11-fold higher than that of the YN-S population, respectively. However, the I_50_ value for AH-R population was 0.0470 μmol·L^−1^, which was 4.12-fold higher than that of the YN-S, indicating a significant decrease in ALS sensitivity to penoxsulam in the AH-R population. Therefore, resistance in the HLJ-R and JL-R populations to penoxsulam may not be associated with a decrease in ALS sensitivity, whereas the reduced sensitivity of ALS in the AH-R population appears to be one of the key factors contributing to resistance development.

**Table 3:** The sensitivity of *in vitro* ALS extracted from different *Echinochloa crus-galli* populations to penoxsulam.

Populations	Total ALS activity (nmol acetoin mg^−1^, protein min^−1^)	I_50_ (µmol·L^−1^)	RI
JL-R	0.520	0.0118 ± 0.0035	1.04
AH-R	0.289	0.0470 ± 0.0068	4.12
HLJ-R	0.416	0.0126 ± 0.0083	1.11
YN-S	0.260	0.0114 ± 0.0021	1.00

Total ALS activity refers to the ALS enzyme activity measured using water treatment; resistance index (RI) was calculated by dividing the I_50_ value of the resistant population by that of the susceptible population.

### 3.4 *ALS* gene expression

**Figure 6:**
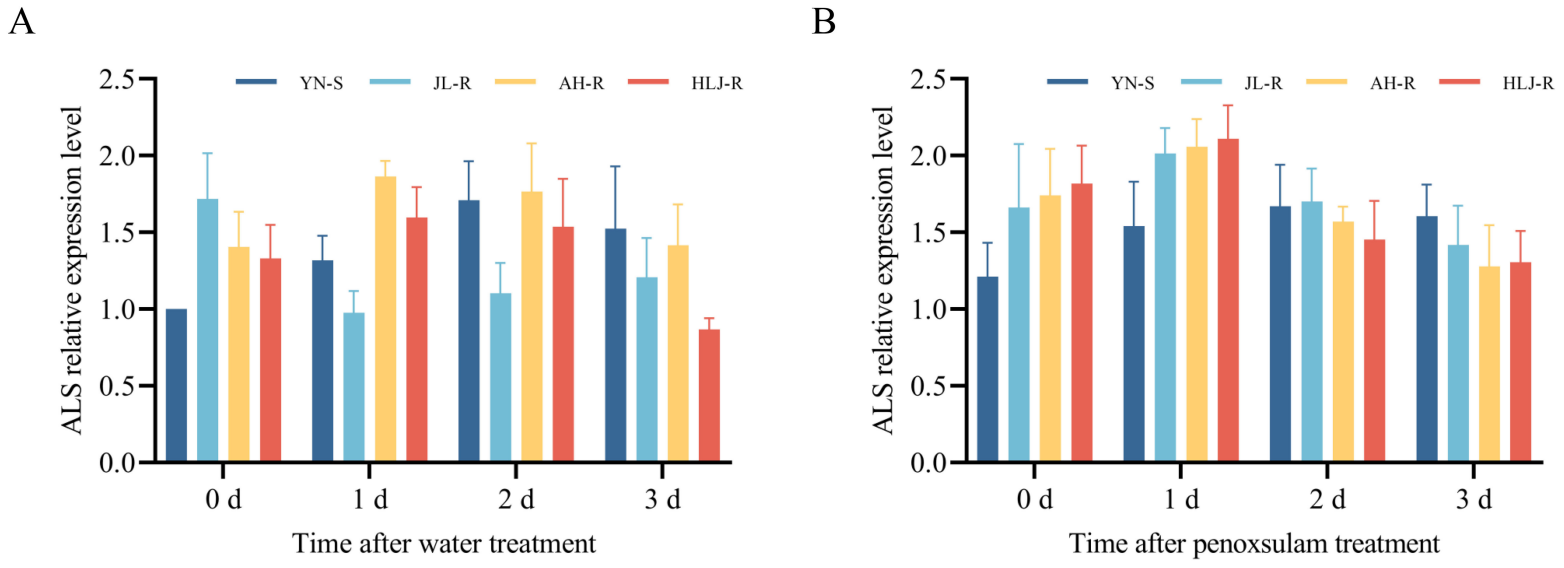
Relative expression levels of the ALS gene in different mutant populations in response to penoxsulam. **(A)** represents treatment with water only, while **(B)** represents treatment with penoxsulam.

In this study, the transcriptional expression differences in *ALS* genes were compared among the resistant populations JL-R (Pro-197-His), AH-R (Trp-574-Leu), HLJ-R (Pro-197-Leu), and the susceptible population YN-S. The results of *ALS* gene expression levels, as shown in **Figures 6A, B**, indicated that there were no significant differences in *ALS* gene expression at 0 d, 1 d, 2 d, and 3 d after water treatment between the resistant populations JL-R, AH-R, HLJ-R, and the susceptible population YN-S (**Figure 6A**). Furthermore, there were no significant differences in *ALS* gene expression among these populations with the treatment of penoxsulam at 15 g a.i. ha^−1^ (**Figure 6B**). Therefore, the resistance to penoxsulam in JL-R, AH-R, and HLJ-R populations appears to be unrelated to the transcriptional level of *ALS* gene.

### 3.5 Homology modeling and molecular docking

To gain a deeper understanding of the potential mechanism driving the differences in herbicide binding specificity to penoxsulam between the non-mutated and the three mutated ALS proteins in *E. crus-galli*, homology models of the ALS proteins were constructed, and comparative structural analyses were performed. The template protein 3e9y exhibited the highest sequence identity with the target ALS proteins from *E. crus-galli*, at approximately 75.00% (**Table 4**). The results of the Ramachandran plot indicated that more than 99% of the amino acid residues in the five different target ALS proteins were situated in the most favored and additional allowed regions (**Table 5**; **Supplementary Figure S1**). This validation confirmed that the protein model structures were of high quality and could be reliably used for subsequent molecular docking studies.

**Table 4:** Comprehensive evaluation of protein structure models generated by Swiss model.

Template ID	Target protein	Sequence identity (%)	GMQE	QMEANDisCo Global
3e9y	YN-S_ALS1_643AA	75.00	0.84	0.86 ± 0.05
	JL-R_ALS1_643AA	74.83	0.84	0.86 ± 0.05
	AH-R_ALS1_643AA	74.96	0.84	0.86 ± 0.05
	YN-S_ALS2_644AA	75.00	0.84	0.86 ± 0.05
	HLJ-R_ALS2_644AA	75.13	0.83	0.86 ± 0.05

GMQE (Global Model Quality Estimation) is a metric used to estimate the overall quality of a protein structure model. Higher GMQE scores imply greater confidence in the accuracy of the model, considering factors like sequence identity, template resolution, and target sequence coverage; QMEANDisCo Global is a quality assessment metric within the QMEAN framework, evaluating the overall quality of protein structure models. A higher QMEANDisCo Global score signifies better agreement between the model and experimental data, indicating higher overall model quality.

**Table 5:** The data of Ramachandran plot analysis of the five different target ALS proteins in *E. crus-galli*.

Target protein	Residues in most favored regions (%)	Residues in additional allowed regions (%)	Residues in generously allowed regions (%)	Residues in disallowed regions (%)
YN-S_ALS1_643AA	89.5	10.1	0.4	0.0
JL-R_ALS1_643AA	89.5	10.1	0.2	0.2
AH-R_ALS1_643AA	89.7	9.9	0.4	0.0
YN-S_ALS2_644AA	89.5	10.1	0.4	0.0
HLJ-R_ALS2_644AA	89.5	10.1	0.4	0.0

**Figure 7:**
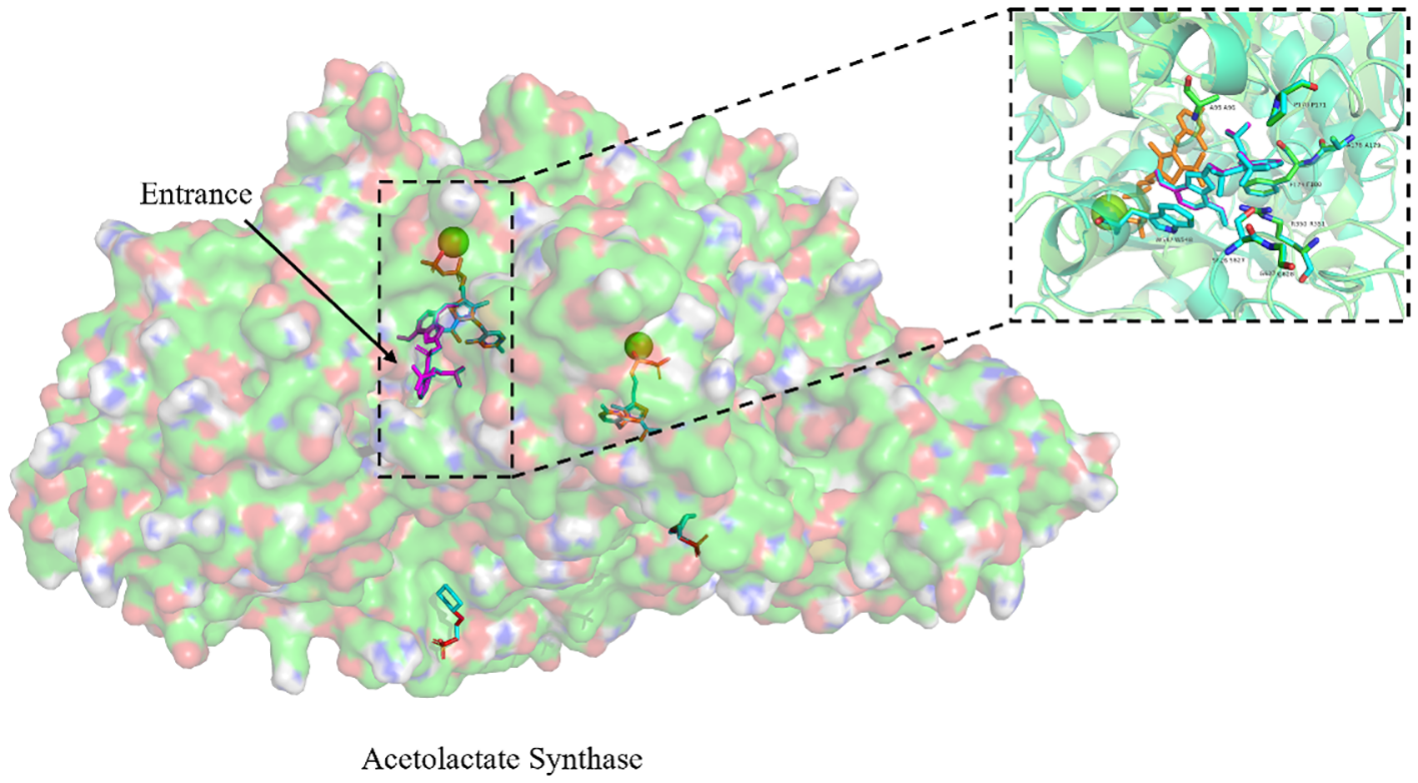
Exploring the binding conformation of penoxsulam with ALS proteins (YN-S_ALS1_643AA and YN-S_ALS2_644AA) in *E. crus-galli* through molecular docking. The green spheres represent Mg^2+^, the orange ligand represents FAD, and the binding positions of penoxsulam molecules in YN-S_ALS1_643AA and YN-S_ALS2_644AA proteins are consistent. Amino acids in the vicinity of the binding site are named based on the *Arabidopsis thaliana* ALS sequence for localization.

**Figure 8:**
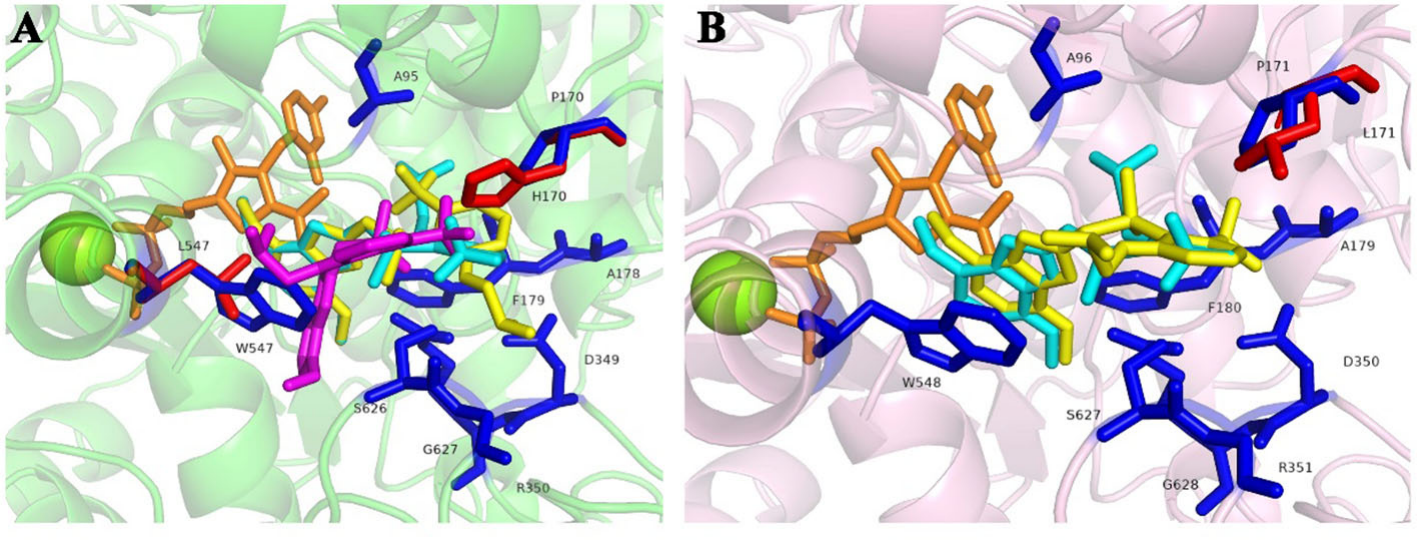
Molecular docking conformation of penoxsulam and ALS proteins in *E*. *crus-galli*: **(A)** The docking conformation of penoxsulam and three ALS1_643AA proteins: the residues of YN-S_ALS1_643AA are represented in blue, and the docking interaction with penoxsulam appeared in a peacock blue shade. The residues of JL-R_ALS1_643AA are the same as YN-S_ALS1_643AA, except for H170 highlighted in red, and the docking interaction with penoxsulam is shown in yellow. The residues of AH-R_ALS1_643AA are the same as YN-S_ALS1_643AA, except for L547 highlighted in red, and the docking interaction with penoxsulam is shown in purple. **(B)** The docking conformation of penoxsulam and two ALS2_644AA proteins: the residues of YN-S_ALS2_644AA are represented in blue, and the docking interaction with penoxsulam appears in a peacock blue shade. The residues of HLJ-R_ALS2_644AA are the same as YN-S_ALS2_644AA, except for H171 highlighted in red, and the docking interaction with penoxsulam is shown in yellow.

**Figure 9:**
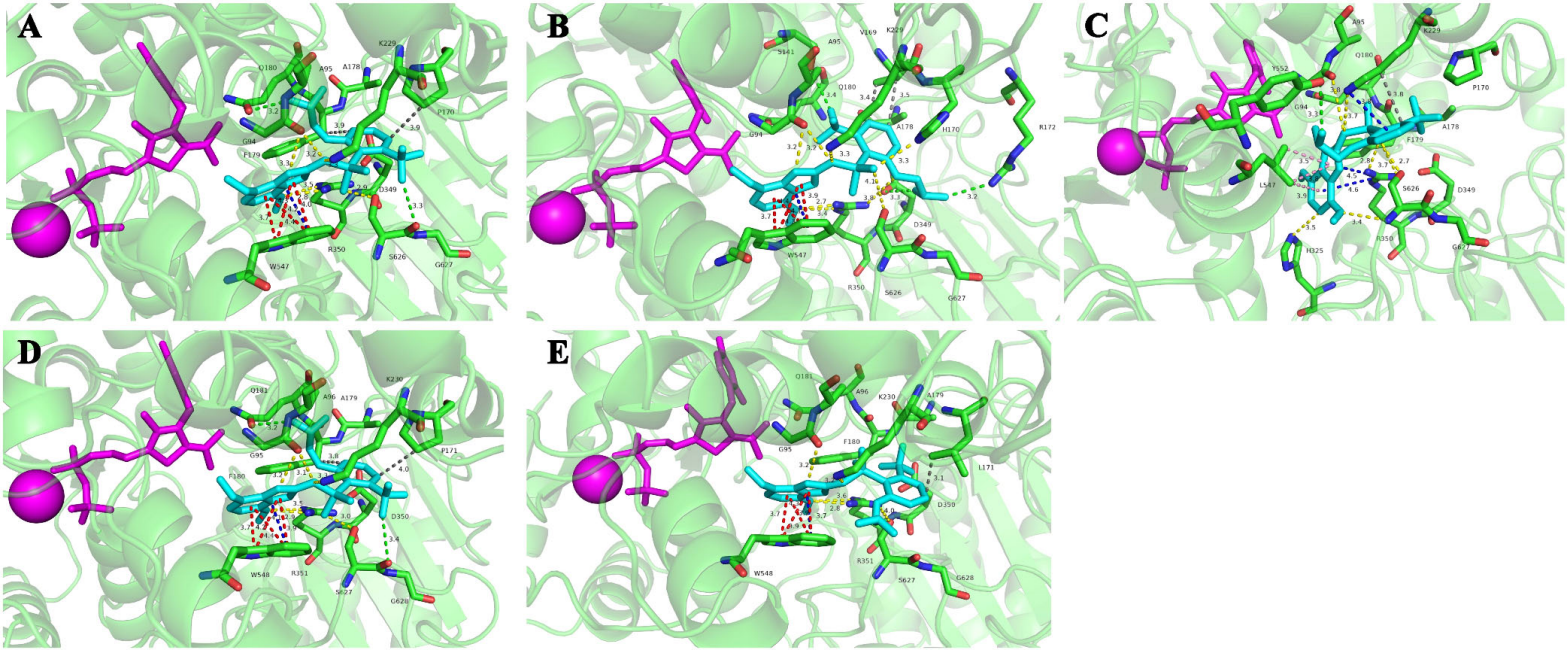
Molecular docking conformations of the mutated ALS proteins, Pro-197-His/Leu, and Trp-574-Leu, alongside the non-mutated *E crus-galli* ALS protein, to penoxsulam. The purple spherical ligand and small molecule ligand represent Mg^2+^ and FAD, respectively. Penoxsulam is depicted in peacock blue by stick model, and the amino acid residues interacting with penoxsulam are named using the single-letter amino acid code followed by the residue number, such as Pro170 written as P170. **(A)** The docking simulation conformation of penoxsulam with YN-S_ALS1_643AA. **(B)** The docking simulation conformation of penoxsulam with JL-R_ALS1_643AA. **(C)** The docking simulation conformation of penoxsulam with AH-R_ALS1_643AA. **(D)** The docking simulation conformation of penoxsulam with YN-S_ALS2_644AA. **(E)** The docking simulation conformation of penoxsulam with HLJ-R_ALS2_644AA. Gray line: hydrophobic interaction. Yellow line: hydrogen bond. Red line: π–π stacking interaction. Blue line: π–cation interaction. Green line: halogen bond. Pink line: π–sigma interaction.

The homology modeling of ALS proteins included six ligands: two FAD molecules, two magnesium ions (Mg^2+^), and two 2-(cyclohexylamino)ethanesulfonic acid (CHE) molecules. Molecular docking simulations demonstrated consistent binding interactions of penoxsulam with two non-mutated ALS proteins, YN-S_ALS1_643AA and YN-S_ALS2_644AA with binding energies of −7.5 kcal mol^−1^ and −7.3 kcal mol^−1^, respectively (**Table 6**). The binding site of penoxsulam was situated close to the FAD-binding channel within the two subunits of *E. crus-galli* ALS. In this binding mode, the triazole-pyrimidine ring inserted into the interior of the channel, while the benzene ring structure was located on the outer surface of the channel (**Figure 7**). However, mutations occurred at H170 (equivalent to *A. thaliana* H197) in the *E. crus-galli* ALS1 and L171 (equivalent to *A. thaliana* L197) in the *E. crus-galli* ALS2, resulting in changes to the docking mode of penoxsulam with ALS1 and ALS2, with binding energies of −6.3 kcal mol^−1^ and −6.7 kcal mol^−1^, respectively (**Table 6**; **Figure 8A**). An increase in binding energy values indicates a reduced affinity of the ALS protein for penoxsulam. Specifically, the H170 mutation resulted in the formation of hydrogen bonds between the H170 residue and the benzene ring, disrupting pre-existing hydrophobic interactions. The penoxsulam benzene ring and sulfonyl group rotated, leading to changes in interactions with residues like F179, V169, and S626 (equivalent to *A. thaliana* F206, V196, and S653) (**Figures 8A**, **9A, B**). Similarly, the ALS2_L171 mutation resulted in changes in the hydrophobic interactions involving the P171 residue in ALS2, leading to alterations in the binding conformation of penoxsulam with ALS2 and changes in interactions with surrounding amino acid residues (**Figures 8B**, **9D, E**). Additionally, the mutation at L547 (equivalent to *A. thaliana* L574) in *E. crus-galli* ALS1 resulted in a binding energy of −7.2 kcal mol^−1^ with penoxsulam. The ALS1_L547 mutation disrupted the π–π stacking interaction between W547 and the pyrimidine ring, leading to altered interactions between penoxsulam and the surrounding residues. Notably, the pyrimidine ring of the penoxsulam molecule underwent a significant rotation (**Figures 8A**, **9C**).

**Table 6:** The results of 20 independent molecular docking by Autodock Vina.

Target protein	Affinity (kcal/mol)	Distance from best mode (RMSD)
YN-S_ALS1_643AA	−7.5	3.551
JL-R_ALS1_643AA	−6.3	3.612
AH-R_ALS1_643AA	−7.2	3.309
YN-S_ALS2_644AA	−7.3	3.533
HLJ-R_ALS2_644AA	−6.7	2.762

RMSD refers to the root mean square deviation from the best mode in the Vina results.

### 3.6 Heterologous expression of ALS protein and activity to penoxsulam

The recombinant ALS proteins were expressed by the Bac-to-Bac baculovirus expression system with His-tags. The theoretical molecular weight of ALS1 and ALS2 is approximately 69 kDa. Western blot analysis revealed a distinct band approximately 72 kDa, confirming the successful expression of the ALS proteins. The results of purified ALS protein activity to penoxsulam are presented in **Table 7**. When treated with 1 μmol·L^−1^ penoxsulam, the various ALS proteins exhibited distinct responses. Specifically, WT_ALS1_643AA and WT_ALS2_644AA displayed significant inhibition, with ALS activity inhibition rates of 86.81% and 79.26%, respectively. In contrast, ALS proteins with different mutations showed considerably lower activity inhibition rates compared to non-mutated ALS proteins. Notably, the ALS protein with the Trp-574-Leu mutation exhibited the lowest level of inhibition, consistent with our previous *in vitro* ALS enzyme activity results.

**Table 7:** The activity assay of ALS protein expressed via baculovirus system in response to penoxsulam.

Population	ALS activity with 1μmol·L^−1^ penoxsulam (nmol acetoin mg^−1^, protein min^−1^)	The inhibition of ALS activity (%)
197-His_ALS1_643AA	0.340	42.86^*^
574-Leu_ALS1_643AA	0.395	35.40^*^
197-Leu_ALS2_644AA	0.235	48.25^*^
WT_ALS1_643AA	0.099	86.81
WT_ALS2_644AA	0.144	79.26

^*^ Independent samples t-test was employed for data analysis, indicating significant differences compared to the control (*p* < 0.05).

## 4 Discussion

A single mutation in the target enzyme is regarded as the most common cause of resistance evolution to ALS inhibiting herbicides. So far, there are 12 types of mutations documented in ALS inhibitors in *Echinochloa* spp. In the present study, three types of mutations (Pro-197-His, Trp-574-Leu, and Pro-197-Leu) were confirmed from three *E. crus-galli* populations; Pro-197-Leu and Trp-574-Leu mutations have been previously reported, and Pro-197-His mutation was first detected in *E. crus-galli.*

Mutations in the target enzyme can induce structural changes in its spatial conformation, leading to impaired or weakened binding between the target enzyme and herbicides (Duggleby et al., 2008; Yu and Powles, 2014). The point mutations in the target site of action are widely reported TSR mechanisms. For instance, the Asp-2078-Glu mutation in acetyl-coenzyme A carboxylase (ACCase) conferred resistance to ACCase herbicides in *E. crus-galli* (Fang et al., 2020), the Pro-197, Asp-376, and Trp-574 mutations in ALS conferred resistance to ALS inhibitors in *D. sophia* L (Deng et al., 2017; Wang et al., 2021; Yang et al., 2018). As widely recognized, *E. crus-galli*, a hexaploid plant, possesses at least three copies of the *ALS* gene in its genome. Following the sequencing of these three distinct *ALS* copies, three types of mutations were identified in the JL-R, AH-R, and HLJ-R populations (**Figure 4**). According to our knowledge, the Pro-197-His mutation in *ALS1* was detected in *E. crus-galli* for the first time. This specific mutation has been demonstrated to confer resistance to ALS inhibitors in both *D. sophia* (Deng et al., 2015) and *Galium aparine* var. Tenerum Gren.et (Godr.) Rebb (Deng et al., 2019). This study found that *E. crus-galli* with the ALS-197-His/Leu mutation exhibited moderate-level resistance to penoxsulam. In contrast, *Cyperus difformis* L. with the ALS-197-His mutation and *Amaranthus retroflexus* L. with the ALS-197-Leu mutation displayed high levels of resistance to TPs inhibitors (Sibony et al., 2001; Tehranchian et al., 2015). These findings suggest that research on ALS mutations should be specific to the weed species involved. Therefore, we conducted an in-depth investigation of the newly identified mutations in *E. crus-galli*.

The overexpression of the target enzyme gene is considered one of the mechanisms in TSR. Panozzo et al. found that the expression levels of mutated *ALS* gene copies in *E. crus-galli* and *E. oryzicola* were significantly higher than that of non-mutated *ALS* gene copies (Panozzo et al., 2021). Additionally, *ACCase* gene overexpression was shown to confer resistance to ACCase inhibitors in *Digitaria sanguinalis* L (Laforest et al., 2017). However, there is no direct evidence of a correlation between the expression level and mutations in the *ALS* gene. In a population of *Capsella bursa-pastoris* L. Medik. with Pro-197-Ser and Pro-197-His mutations in the *ALS* gene copies, Wang et al. discovered that the *ALS* gene expression level did not significantly differ from that of the sensitive population (Wang et al., 2019). Our study conducted differential expression analysis on *ALS* genes and found no significant differences in expression between *ALS* genes in populations with different mutations (Pro-197-His, Trp-574-Leu, and Pro-197-Leu) and *ALS* genes in the sensitive population. This suggests that the resistance of the JL-R, AH-R, and HLJ-R populations to penoxsulam was not related to the overexpression of the *ALS* gene.

The determination of ALS enzyme activity through the colorimetric reaction of creatine and α-naphthol with 2-aceto-2-hydroxybutyrate is the most commonly used method in ALS *in vitro* enzyme assays (Fang et al., 2022; Yang et al., 2018). *In vitro* ALS enzyme activity analysis provides a rapid means of identifying weed resistance to ALS inhibitors. Cao et al. demonstrated that a reduced sensitivity of the ALS enzyme to imazethapyr was a crucial factor in conferring resistance to *Chenopodium album* L. against imazethapyr (Cao et al., 2022). In this study, the ALS *in vitro* enzyme activities of purified F1 generation plants from JL-R, AH-R, and HLJ-R were analyzed. The present research revealed that the I_50_ value of the AH-R (Trp-574-Leu) population was 4.12 times higher than that of the sensitive population, indicating that the decreased sensitivity of the ALS in the AH-R population was one of the reasons for its resistance to penoxsulam. The I_50_ values of the JL-R (Pro-197-His) and HLJ-R (Pro-197-Leu) populations exhibited no significant differences compared to the sensitive population. The populations of JL-R and HLJ-R exhibited moderate resistance to penoxsulam, with mutations occurring solely in one copy of the ALS gene. This observation may account for the lack of significant differences in their ALS *in vitro* enzyme activity.

Homology modeling and molecular docking techniques were extensively used in previous studies of protein–small molecule interactions (Joshi, 2016; Palma-Bautista et al., 2022; Zhao et al., 2022). The mutation of amino acids at specific positions in ALS has been verified to modify the binding forces between ALS and inhibitors, resulting in ALS resistance. For example, the double *ALS* gene mutation (Pro-197-Ser plus Trp-574-Leu) in *C. bursa-pastoris* induced alterations in H-bond, π–π, and π–sulfur interactions between ALS and herbicide, leading to high resistance to mesosulfuron-methyl (Lu et al., 2023). Similarly, the ALS mutation at position 206 (Phe-206-Leu) has been confirmed to confer penoxsulam resistance in *E. crus-galli* due to the disappearance of π–π interaction between the 206 site position and penoxsulam (Fang et al., 2022). ALS consists of catalytic and regulatory subunits, with the latter providing feedback inhibition (Duggleby et al., 2008). ALS inhibitors generally do not directly bind to the catalytic site; instead, they bind to the region located at the entrance of the ALS active site channel, where the Pro-197 and Trp-574 sites are situated (McCourt et al., 2006). The side chain of proline at the Pro-197 site is a saturated hydrocarbon, and this saturated hydrocarbon side chain imposes certain restrictions on the spatial structure of the protein. Liu and coworkers used molecular docking to discover that the Pro-197-Ser mutation in the ALS of *E. phyllopogon* confers resistance to various ALS inhibitors (Liu et al., 2019). This resistance is attributed to changes in the spatial structure of the substrate center due to the variation of the residue at the Pro-197 site, resulting in resistance to TPs, SUs, and SCTs, while remaining sensitive to PTBs and IMIs (Liu et al., 2019). In this study, we found that penoxsulam bound to *E. crus-galli* ALS by inserting the double heterocyclic ring of triazole and pyrimidine into the ALS channel. The π–π stacking formed by the aromatic rings of Phe-206 and Trp-574 with the double heterocyclic ring of penoxsulam was disrupted by the Trp-574-Leu mutation. The Trp-574-Leu mutation impacted contacts of surrounding residues with penoxsulam. This disruption reduced the binding strength between penoxsulam and ALS, consistent with previous research (Fang et al., 2022). Additionally, Trp-574 served as the key residue defining the shape of the substrate access tunnel, and its mutation to another residue was expected to significantly weaken the binding of IMIs (Duggleby et al., 2008). In the present study, although the binding energy between the ALS1 protein and penoxsulam was not significantly reduced following the Trp-574-Leu mutation, the smaller molecular weight of Leu compared to Trp enlarged the binding pocket at the channel entrance. As a result, the inhibitor could not effectively prevent the substrate from reaching the active site, leading to resistance to penoxsulam. Mutations at the Pro-197 site to Leu and His modify the spatial structure of this region due to changes in the amino acid side chains. This alteration may hinder the ability of herbicides to enter the binding channel. Although the affinities of the ALS protein with the Pro-197-His and Pro-197-Leu mutations for penoxsulam are lower than that of the Trp-574-Leu mutated ALS protein, the binding mode of penoxsulam remains largely unchanged. Consequently, it continues to effectively obstruct the substrate from accessing the active site, resulting in a lower level of resistance to penoxsulam compared to the ALS protein with the Trp-574-Leu mutation.

The previous study revealed that the impact of resistant alleles could diminish with the increase in the percentage of susceptible allele transcripts in polyploid weeds (Iwakami et al., 2012). In this current study, the expression of mutated ALS alleles might have experienced a dilution effect from sensitive alleles, potentially reducing the contribution to resistance. Therefore, the Bac-to-Bac baculovirus expression system was employed to individually express the mutant ALS protein and analyze its activity with penoxsulam. This system allows cells to grow in suspension, facilitating large-scale cultivation and demonstrating the capability for simultaneous expression of multiple genes with enhanced protein modification processing abilities (Fang et al., 2022; Van Oers et al., 2015). In this study, we successfully obtained wild-type (WT) and three mutant ALS proteins. Upon treatment with penoxsulam, the results revealed that, compared to the WT ALS proteins (WT_ALS1_643AA and WT_ALS2_644AA), the activity inhibition rates of the three mutant ALS proteins (197-His_ALS1_643AA, 574-Leu_ALS1_643AA, and 197-Leu_ALS2_644AA) were reduced at 1 μmol·L^−1^ penoxsulam. This finding was consistent with our differential analysis of ALS genes and molecular docking results, providing protein-level evidence that mutations in *E. crus-galli* ALS (Trp-574-Leu, Pro-197-Leu, and Pro-197-His) induce resistance to penoxsulam. Additionally, this also explained the impact of the dilution effect of sensitive allele genes in the *in vitro* detection of mutant plant ALS activity.

The ALS inhibitor penoxsulam, developed by Dow AgroSciences, is a trizolopyrimidine herbicide applied in paddy fields with the wide herbicidal spectrum. It has great effect not only on aquatic weed control but also on *E. crus-galli*, which has resistance to herbicides like quinclorac, propanil, and sulfonylurea. It was registered in China in 2007 and developed as a key product in weeds control in paddy field since then. However, the overreliance on the herbicide has led to the development of resistant weeds related to TSR and NTSR (Fang et al., 2019a, Fang et al., 2022, Fang et al., 2019b; Feng et al., 2022). In the past decade, *E. crus-galli* has been reported to evolve resistance to several sites of action of herbicides (Fang et al., 2019a, Fang et al., 2022, Fang et al., 2019b; Pan et al., 2022; Sun et al., 2023; Vázquez-García et al., 2021). Under the continuous selective pressure of herbicides, herbicide resistance evolution in weeds has become an inevitable consequence. Mutations in the target site of action in weeds frequently result not only in resistance to specific herbicides but also in cross-resistance to several herbicides (Han et al., 2012; Singh et al., 2019; Vital Silva et al., 2022). Through interspecies cross-pollination, mutation genes swiftly disseminate within the species. Therefore, there is an urgent need for exploring the mechanisms of weed resistance and the formulation of innovative, sustainable weed management methods to effectively delay and manage the evolution of herbicide resistance (Beckie et al., 2019; Pan et al., 2022).

## Data Availability

The original contributions presented in the study are included in the article/**Supplementary Material**. Further inquiries can be directed to the corresponding author.
