# Supplementary material for: Trp-574-Leu and the novel Pro-197-His/Leu mutations contribute to penoxsulam resistance in Echinochloa crus-galli (L.) P. Beauv
Source: Front Plant Sci. 2024 Nov 25;15:1488976. doi: 10.3389/fpls.2024.1488976 (PMC11625580; doi:10.3389/fpls.2024.1488976)
Supplement: Supplementary file 1 [file Table1.docx]

Supplementary Material

Table S1 Amino acid sequences for protein models

| Target ALS protein | Amino acid sequence |
| --- | --- |
| YN-S_ALS1_643AA | MATTAAAAATALTGATTAAPRPRRRAYSASARRAALAAPIRCSAASPAAPTPALAPPATPLRPWGPTEPRKGADILVEALERCGVRDVFAYPGGASMEIHQALTRSPVIANHLFRHEQGEAFAASGFARSSGRVGVCVATSGPGATNLVSALADALLDSIPMVAITGQVPRRMIGTDAFQETPIVEVTRSITKHNYLVLDIDDIPRVVQEAFFLASSGRPGPVLVDIPKDIQQQMAVPVWNTPMSLPGYIARLPKPPATELLEQVLRLVGESRRPVLYVGGGCAASGEELCRFVEMTGIPVTTTLMGLGNFPSDDPLSLRMLGMHGTVYANYAVDKADLLLAFGVRFDDRVTGKIEAFASRAKIVHIDIDPAEIGKNKQPHVSICADVKLALQGMNALLEGIISKKSFDFGSWHDELDQQKREFPLGYKTFDEEIQPQYAIQVLDELTKGEAIIATGVGQHQMWAAQYYTYKRPRQWLSSAGLGAMGFGLPAAAGAAVANPGVTVVDIDGDGSFLMNIQELAMIRIENLPVKVFVLNNQHLGMVVQWEDRFYKANRAHTYLGNPENESEIYPDFVMIAKGFNIPAVRVTKKSEVRAAIKKMLETPGPYLLDIIVPHQEHVLPMIPSGGAFKDMILDGDGRTVY* |
| JL-R_ALS1_643AA | MATTAAAAATALTGATTAAPRPRRRAYSASARRAALAAPIRCSAASPAAPTPALAPPATPLRPWGPTEPRKGADILVEALERCGVRDVFAYPGGASMEIHQALTRSPVIANHLFRHEQGEAFAASGFARSSGRVGVCVATSGPGATNLVSALADALLDSIPMVAITGQVHRRMIGTDAFQETPIVEVTRSITKHNYLVLDIDDIPRVVQEAFFLASSGRPGPVLVDIPKDIQQQMAVPVWNTPMSLPGYIARLPKPPATELLEQVLRLVGESRRPVLYVGGGCAASGEELCRFVEMTGIPVTTTLMGLGNFPSDDPLSLRMLGMHGTVYANYAVDKADLLLAFGVRFDDRVTGKIEAFASRAKIVHIDIDPAEIGKNKQPHVSICADVKLALQGMNALLEGIISKKSFDFGSWHDELDQQKREFPLGYKTFDEEIQPQYAIQVLDELTKGEAIIATGVGQHQMWAAQYYTYKRPRQWLSSAGLGAMGFGLPAAAGAAVANPGVTVVDIDGDGSFLMNIQELAMIRIENLPVKVFVLNNQHLGMVVQWEDRFYKANRAHTYLGNPENESEIYPDFVMIAKGFNIPAVRVTKKSEVRAAIKKMLETPGPYLLDIIVPHQEHVLPMIPSGGAFKDMILDGDGRTVY* |
| AH-R_ALS1_643AA | MATTAAAAATALTGATTAAPRPRRRAYSASARRAALAAPIRCSAASPAAPTPALAPPATPLRPWGPTEPRKGADILVEALERCGVRDVFAYPGGASMEIHQALTRSPVIANHLFRHEQGEAFAASGFARSSGRVGVCVATSGPGATNLVSALADALLDSIPMVAITGQVPRRMIGTDAFQETPIVEVTRSITKHNYLVLDIDDIPRVVQEAFFLASSGRPGPVLVDIPKDIQQQMAVPVWNTPMSLPGYIARLPKPPATELLEQVLRLVGESRRPVLYVGGGCAASGEELCRFVEMTGIPVTTTLMGLGNFPSDDPLSLRMLGMHGTVYANYAVDKADLLLAFGVRFDDRVTGKIEAFASRAKIVHIDIDPAEIGKNKQPHVSICADVKLALQGMNALLEGIISKKSFDFGSWHDELDQQKREFPLGYKTFDEEIQPQYAIQVLDELTKGEAIIATGVGQHQMWAAQYYTYKRPRQWLSSAGLGAMGFGLPAAAGAAVANPGVTVVDIDGDGSFLMNIQELAMIRIENLPVKVFVLNNQHLGMVVQLEDRFYKANRAHTYLGNPENESEIYPDFVMIAKGFNIPAVRVTKKSEVRAAIKKMLETPGPYLLDIIVPHQEHVLPMIPSGGAFKDMILDGDGRTVY* |
| YN-R_ALS2_644AA | MATTAAAAATALTGATTAAPRPRRRAYSASARRAALAAPIRCSAASPAAPTPALAPPATPLRPWGPTEPRKGADILVEALERCGVRDVFAYPGGASMEIHQALTRSPVIANHLFRHEQGEAFAASGFARSSGRVGVCVATSGPGATNLVSALADALLDSIPMVAITGQVPRRMIGTDAFQETPIVEVTRSITKHNYLVLDIDDIPRVVQEAFFLASSGRPGPVLVDIPKDIQQQMAVPVWNTPMSLPGYIARLPKPPATELLEQVLRLVGESRRPVLYVGGGCAASGEELCRFVEMTGIPVTTTLMGLGNFPSDDPLSLRMLGMHGTVYANYAVDKADLLLAFGVRFDDRVTGKIEAFASRAKIVHIDIDPAEIGKNKQPHVSICADVKLALQGMNALLEGIISKKSFDFGSWHDELDQQKREFPLGYKTFDEEIQPQYAIQVLDELTKGEAIIATGVGQHQMWAAQYYTYKRPRQWLSSAGLGAMGFGLPAAAGAAVANPGVTVVDIDGDGSFLMNIQELAMIRIENLPVKVFVLNNQHLGMVVQWEDRFYKANRAHTYLGNPENESEIYPDFVMIAKGFNIPAVRVTKKSEVRAAIKKMLETPGPYLLDIIVPHQEHVLPMIPSGGAFKDMILDGDGRTVY* |
| HJL-R_ALS2_644AA | MATTAAAAATAATAAAALTGATTAAPRPSRRGYSAAAARRAAPIRCSAASPATATAPPATPLRPWGPTEPRKGADILVEALERCGVRDVFAYPGGASMEIHQALTRSPVIANHLFRHEQGEAFAASGFARSSGRVGVCVATSGPGATNLVSALADALLDSIPMVAITGQVLRRMIGTDAFQETPIVEVTRSITKHNYLVLDIDDIPRVIQEAFFLASSGRPGPVLVDIPKDIQQQMAVPVWNTPMSLPGYIARLPKPPATELLEQVLRLVGESRRPVLYVGGGCAASGEELRRFVEMTGIPVTTTLMGLGNFPSDDPLSLRMLGMHGTVYANYAVDKADLLLAFGVRFDDRVTGKIEAFASRAKIVHIDIDPAEIGKNKQPHVSICADVKLALQGMNALLEGIISKKSFDFGSWQDELDQQKREFPLGYKTFDEEIQPQYAIQVLDELTKGEAIIATGVGQHQMWAAQYYTYKRPRQWLSSAGLGAMGFGLPAAAGAAVANPGVTVVDIDGDGSFLMNIQELAMIRIENLPVKVFVLNNQHLGMVVQWEDRFYKANRAHTYLGNPENESEIYPDFVTIAKGFNIPAVRVTKKSEVRAAIKKMLETPGPYLLDIIVPHQEHVLPMIPSGGAFKDMILDGDGRTVY* |


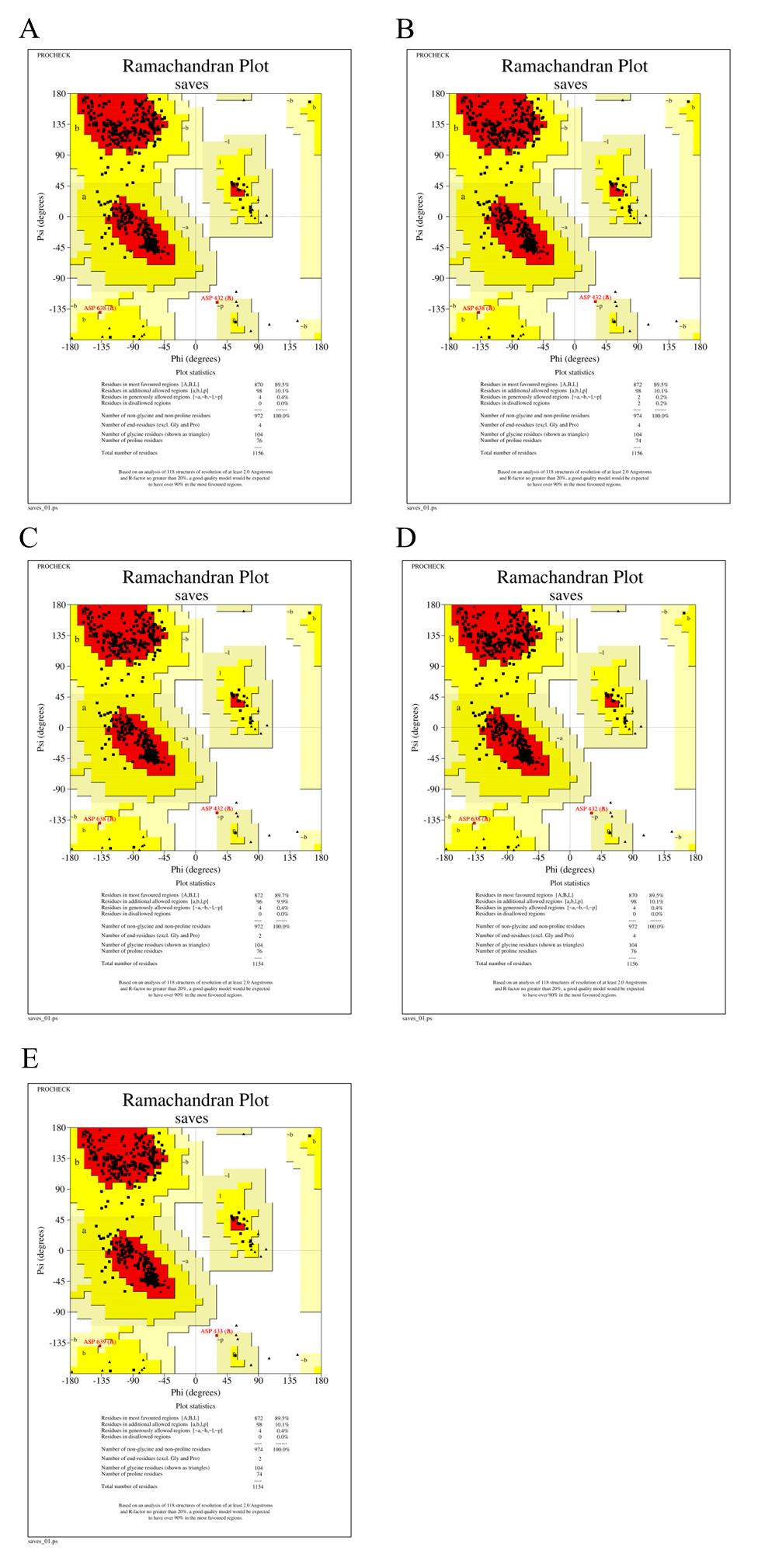


Figure S1 Ramachandran plot analysis of ALS protein models (A, YN-S_ALS1_643AA; B, JL-R_ALS1_643AA; C, AH-R_ALS1_643AA; D, YN-S_ALS2_644AA; E, HLJ-R_ALS2_644AA)
